# Regional brain volume predicts response to methylphenidate treatment in individuals with ADHD

**DOI:** 10.1186/s12888-021-03040-5

**Published:** 2021-01-11

**Authors:** Jung-Chi Chang, Hsiang-Yuan Lin, Junglei Lv, Wen-Yih Issac Tseng, Susan Shur-Fen Gau

**Affiliations:** 1 Department of Psychiatry, National Taiwan University Hospital, Taipei, Taiwan; 2 Department of Psychiatry, National Taiwan University Hospital, Hsin-Chu Branch, Hsin-Chu, Taiwan; 3 Graduate Institute of Clinical Medicine, College of Medicine, National Taiwan University, Taipei, Taiwan; 4 Azrieli Adult Neurodevelopmental Centre and Adult Neurodevelopment and Geriatric Psychiatry Division, Centre for Addiction and Mental Health, Toronto, Ontario Canada; 5 Department of Psychiatry, University of Toronto, Toronto, Ontario Canada; 6 Sydney Imaging and School of Biomedical Engineering, University of Sydney, Camperdown, NSW Australia; 7 Graduate Institute of Brain and Mind Sciences, National Taiwan University College of Medicine, Taipei, Taiwan; 8 Institute of Medical Device and Imaging, National Taiwan University College of Medicine, Taipei, Taiwan; 9 Department of Psychiatry, College of Medicine, National Taiwan University, Taipei, Taiwan

**Keywords:** ADHD, Methylphenidate, VBM, Striatum, Support vector machine, Treatment response

## Abstract

**Background:**

Despite the effectiveness of methylphenidate for treating ADHD, up to 30% of individuals with ADHD show poor responses to methylphenidate. Neuroimaging biomarkers to predict medication responses remain elusive. This study characterized neuroanatomical features that differentiated between clinically good and poor methylphenidate responders with ADHD.

**Methods:**

Using a naturalistic observation design selected from a larger cohort, we included 79 drug-naive individuals (aged 6–42 years) with ADHD without major psychiatric comorbidity, who had acceptable baseline structural MRI data quality. Based on a retrospective chart review, we defined responders by individuals’ responses to at least one-month treatment with methylphenidate. A nonparametric mass-univariate voxel-based morphometric analysis was used to compare regional gray matter volume differences between good and poor responders. A multivariate pattern recognition based on the support vector machine was further implemented to identify neuroanatomical indicators to predict an individual’s response.

**Results:**

63 and 16 individuals were classified in the good and poor responder group, respectively. Using the small-volume correction procedure based on the hypothesis-driven striatal and default-mode network masks, poor responders had smaller regional volumes of the left putamen as well as larger precuneus volumes compared to good responders at baseline. The machine learning approach identified that volumetric information among these two regions alongside the left frontoparietal regions, occipital lobes, and posterior/inferior cerebellum could predict clinical responses to methylphenidate in individuals with ADHD.

**Conclusion:**

Our results suggest regional striatal and precuneus gray matter volumes play a critical role in mediating treatment responses in individuals with ADHD.

**Supplementary Information:**

The online version contains supplementary material available at 10.1186/s12888-021-03040-5.

## Background

Attention-deficit hyperactivity disorder (ADHD), characterized by impaired attention and impulsivity/behavioral control, is a common neurodevelopmental disorder persisting across the lifespan [1]. Among treatment options, methylphenidate is one of the most common, efficacious, and tolerable psychostimulants as pharmacotherapy for ADHD [2]. Methylphenidate is effective in reducing core symptoms and associated behavioral problems of ADHD, as well as improving academic performance, quality of life, and neuropsychological functions [3, 4]. However, around 30% of individuals with ADHD exhibit poor responses to methylphenidate [5]. Studies indicate that individuals with ADHD having poor intelligence quotient, higher disease severity, and a family history of a psychiatric disorder [6] show a poor response to methylphenidate. Further, higher anxiety levels [7], as well as co-occurring personality, substance use [8], alongside anxiety disorders [9] are related to suboptimal methylphenidate responses in individuals with ADHD.

Investigating an individual’s neurobiological variations may provide better explanations and have translational potentials to help identify those poor responders before the start of methylphenidate prescription. This endeavor is of clinical significance to reduce the suffering of unnecessary drug-related side-effects, the delay from receiving more effective treatment, and the discouragement of the patients and their families. Methylphenidate binds to the dopamine transporter and norepinephrine transporter and blocks their reuptake, thereby increasing the extracellular levels of these neurotransmitters [10]. Earlier pharmacogenetic studies have indicated that certain polymorphisms in norepinephrine [11] or serotonin transporter genes [12], as well as dopamine receptor genes [13] may be associated with responses to methylphenidate. A positron emission tomography study on healthy adults suggests that inter-individual variability in the amount of dopamine released by neurons associates with the degree to which dopamine at synaptic levels increases the following blockade of dopamine transporters by methylphenidate [14]. In individuals with ADHD, methylphenidate also has been shown to increase striatal dopamine availability, which in turn may further affect the corticostriatal systems subserving ADHD symptoms and behaviors related to executive dysfunctions [15]. However, brain phenotypes of individuals with ADHD who likely respond to methylphenidate remain elusive [16].

Among the scarce published reports [16], structural MRI studies yielded mixed findings that individuals with ADHD who are poor responders to methylphenidate appear to have thinner medial frontal lobe [17], smaller corpora callosa white matter (WM) volumes [18], smaller inferior posterior cerebellar volumes, greater caudate volumes and asymmetry [19], and smaller caudate and accumbens volumes concentrations [20]. Most of these studies consisted of limited sample sizes (N of ADHD < 30 in total) [18–20] and did not report or address issues of in-scanner motion. Studies have confirmed that head motion in the MRI scanner would introduce inaccuracy when estimating gray matter (GM) volume and thickness [21, 22]. In addition, most of the studies included participants with major psychiatric comorbidity [17] or with a prior methylphenidate exposure [17]. Co-occurring mental health issues per se have been shown to significantly affect treatment responses to methylphenidate [15]. A meta-analysis of ADHD-associated brain structural alterations revealed that studies with a higher percentage of psychostimulant-treated participants tend to be associated with fewer differences in the striatum (specifically smaller volumes in ADHD) [23]. This suggests that stimulant exposure would affect brain structures in ADHD. Interestingly, the findings from this meta-analysis also indirectly converge to show that structural correlates of responses to psychostimulant might involve the striatum. In sum, despite the inconsistency in directions [19, 20] and methodological caveats, these studies suggest that poor responders are essentially characterized by altered striatal structures, among other mixed findings [16]. Further, functional image studies show frontostriatal connectivity measured by resting-state functional MRI [24], as well as striatal regional cerebral blood flow and its dopamine transporter binding estimated by single-photon emission computed tomography [25] are related to treatment responses in individuals with ADHD.

In addition to the striatal regions and associated corticostriatal circuitries, the default-mode network (DMN) maybe another brain circuitry involved in methylphenidate effects and responses. The DMN comprises the posterior cingulate/precuneus, medial prefrontal cortex, and lateral inferior parietal cortex [26]. It exhibits reduced activity when task-positive networks (mainly the fronto-parieto-striatal circuitries) activate in response to external tasks and is most active when people are engaged in internally-oriented and stimulus-independent cognition [27]. ADHD is characterized by altered DMN connectivity [28] and increased DMN-task-positive network connections [28, 29]. Through increasing dopamine and norepinephrine, methylphenidate has been consistently reported to suppress DMN activities in patients with ADHD while engaging in cognitive tasks (i.e., reducing the DMN-task-positive network connection) [30–33]. Reduction in DMN activity or connectivity with task-positive networks also is associated with methylphenidate-related improvements in core symptoms of individuals with ADHD [34]. The medial prefrontal cortex, which is thinner in poor responders at baseline, as shown in the aforementioned naturalistic study [17], partially corresponds to the one hub region of the DMN. Despite its convergingly essential role in the pathophysiology of ADHD and methylphenidate effects on brain function, the DMN has never been specifically investigated whether its structure and function are related to treatment response to methylphenidate in ADHD.

In this context, this study aimed to characterize the baseline brain structural correlates that distinguished good and poor responders to methylphenidate in medication-naïve patients with ADHD, who did not have major psychiatric comorbidities. Based on the mass-univariate analysis of voxel-based morphometry (VBM), we first employed a combination of both unbiased exploratory whole-brain and hypothesis-driven approaches targeting striatal and DMN regions. Further, we leveraged a multivariate pattern classification method that takes into account interactions between regions, and is capable of making predictions for individual subjects based on brain imaging patterns [35]. This machine learning approach can complement group-level inferences from the preceding mass-univariate analysis [36]. We hypothesized that the ADHD-poor responder group, relative to the good responder group, would have smaller baseline striatal volumes [20, 23]. Taking a typical negative connection relationship between the DMN and frontostriatal task-positive networks [27], as well as a notion that brain structural covariance results from functional connectivity [37], we further hypothesized larger regional GM volumes in the regions within the DMN in the poor responder group at baseline. These brain patterns, among other regional structural information, would provide multivariate indicators to predict an individual’s medication response.

## Methods

### Participants

This study is a *post-hoc* investigation on a uni-center ADHD dataset [29, 38, 39]. All participants with ADHD were clinically referred and recruited from the psychiatric outpatient clinic of National Taiwan University Hospital (NTUH), Taipei, Taiwan. ADHD was clinically diagnosed based on the DSM-IV-TR diagnostic criteria. The clinical diagnoses of ADHD and other psychiatric disorders were further confirmed by semi-structured interviews with the participants and their parents using the Chinese version of the Kiddie Schedule for Affective Disorders and Schizophrenia-Epidemiological version (K-SADS-E) [40–42] for participants younger than 18 years. For those aged 18 years or older, the modified adult version of the ADHD supplement of the K-SADS-E for childhood and current ADHD was administered [4, 43–45]. The severity of ADHD symptoms was also assessed by the parent-rated Swanson, Nolan, and Pelham, Version IV (SNAP-IV) questionnaire (Supplementary Questionnaire) [46–48]. Participants were excluded if they 1) had any systemic medical or major neurological illness; 2) had a past history of major mental health issues, including psychotic disorder, mood disorders, obsessive-compulsive disorder, major anxiety disorders, substance use disorder, autism spectrum disorder; 3) currently had depressive or anxiety symptoms, suicidal ideations; 4) had taken any psychotropic agents, including medications for ADHD; 5) had full-scale IQ < 80 estimated by the Wechsler Intelligence Scale for Children-Third Edition and Wechsler Adult Intelligence Scale-Third Edition [49], respectively, for individuals with an age cutoff at 16 years. Since motor tic, oppositional defiant disorder, and specific phobia are common in participants with ADHD, those with these three comorbidities were not excluded from the studies.

The original studies [29, 38, 39] were approved by the Research Ethics Committee at NTUH (#200903062R, #201204071RIC, #201401024RINC) and registered with ClinicalTrials.gov (NCT00916851, NCT01682915, NCT02642068). The procedures and the purposes were explained face-to-face to the participants and their parents, who then provided the written informed consent. The authors confirm that all methods contributing to this work comply with the ethical standards of the relevant national and institutional guidelines and regulations.

**Fig. 1:**
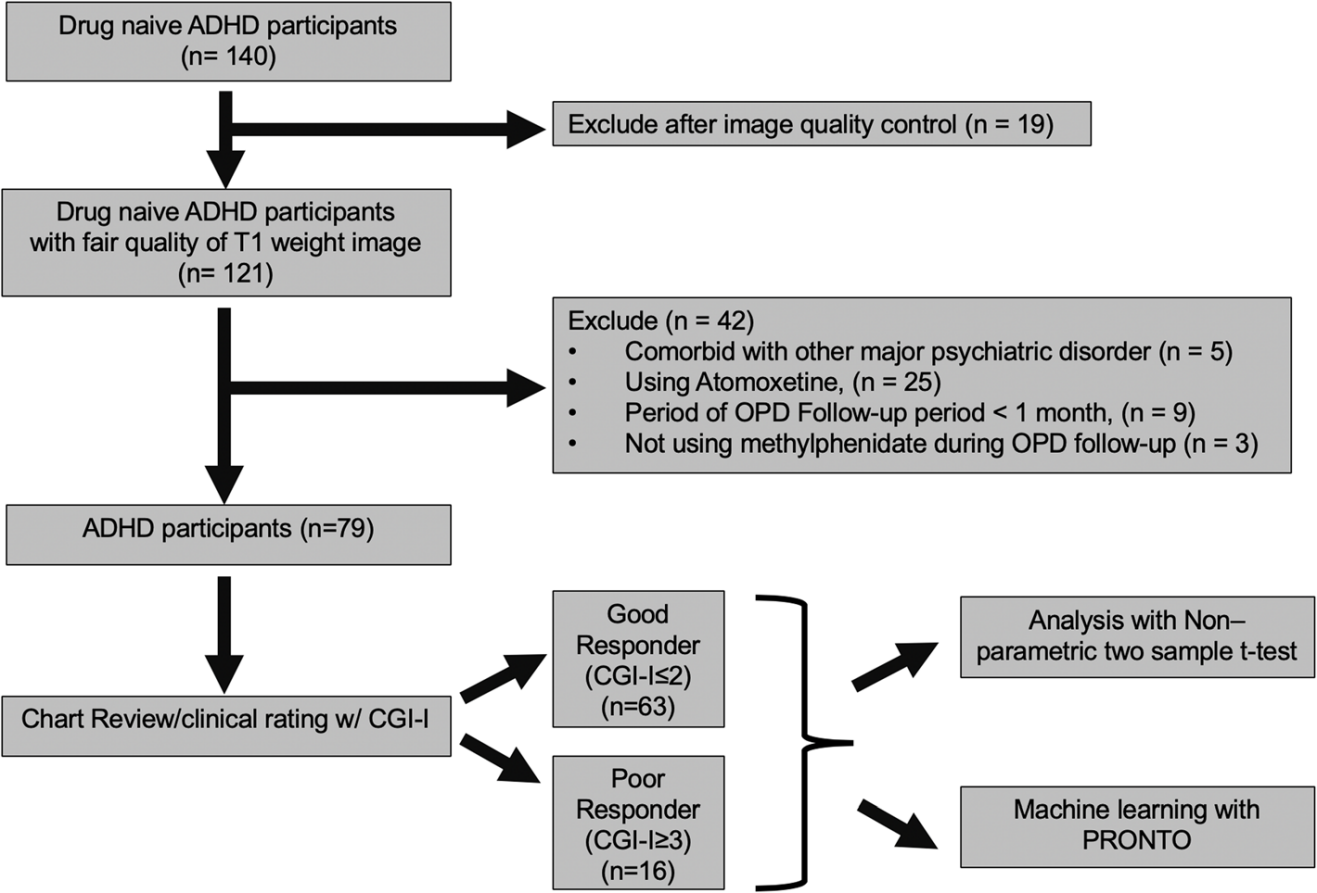
Flow diagram of the procedure. One hundred and forty ADHD participants without previous drug exposure were enrolled initially. Nineteen participants were excluded due to poor quality of T1-weighted images, and 42 individuals were excluded due to comorbidity, received no medication or treatment with atomoxetine, diagnosed with the major psychiatric disorders later, or had the loss to follow-up at the clinic within one month. The final sample of 79 medication-naïve participants with ADHD was divided into good responder group and poor responder groups based on the Clinical Global Impressions–Improvement Scale and then proceeded with further image analysis

All medication-naïve (i.e., never being exposed to methylphenidate or any other psychotropic agent) individuals with ADHD were referred to the studies from NTUH outpatient clinic. If they agreed to be enrolled in the studies [29, 38, 39], they would only start clinically standard treatment until the completion of MRI scans. The present *post-hoc* investigation started with pooling cross-sectional [29, 39] or baseline [38] neuroimaging data from 140 participants with ADHD. Their T1-weighted images were visually inspected (by JCC and HYL) for quality control to ensure data with ratings of “fair” or higher quality based on the Human Connectome Project pipeline [50]. Those with acceptable image data quality were further excluded from the present analysis if they received atomoxetine treatment initially, did not use any medication during follow-up, were diagnosed with those mentioned above major psychiatric disorders later, or had the loss to follow-up at the clinic within 1 month. This step resulted in the final sample of 79 medication-naïve participants with ADHD (age 6–42 years; mean ± SD, 17.50 ± 9.8 years) (Fig. 1).

The assignment of good responder and poor responder was defined by their treatment responses to methylphenidate after at least one-month follow-up in the outpatient clinics by board-certificated senior child psychiatrists. Notably, all participants received immediate-release methylphenidate during this period, as Taiwan’s National Health Insurance can only reimburse this type of formulation initially for people with ADHD who are first-ever treated with psychostimulant. Most of the participants were followed up in the clinics for more than 6 months. The one-month criterion was intentionally decided because a follow-up shorter than this duration only yields limited clinical profiles to evaluate drug responses and does not allow enough time for dose optimization. Two child and adolescent psychiatrists (JCC and HYL) conducted a retrospective chart review. They agreed with each other’s judgment on the final estimation of the patient’s global functioning using the Clinical Global Impressions–Improvement Scale (CGI-I) [51]. The CGI-I is a 7-point scale to compare the patient’s overall clinical condition to the baseline visit. Its scores are rated from “1” for “very much improved” to “7” for “very much worse.” The CGI-I has been broadly used in treatment studies on ADHD in Taiwan [3, 52, 53] and other countries [54–56]. It also shows similar effect sizes in terms of changes with treatment, relative to those derived from the symptom-informed measures in clinical trials of depression [57]. Participants rated ≤2 (“much improved”) on the CGI-I were assigned to the good responder group, while those rated ≥3 (“minimally improved”) were grouped as the poor responder. This cutoff was stringently set, considering the potential bias due to a placebo effect or equivocal documentation in the charts.

### Image acquisition and preprocessing

High-resolution T1-weighted images were acquired by a 3D Magnetization Prepared Rapid Acquisition Gradient Echo sequence on a 3-T MRI scanner (Siemens Magnetom Tim Trio) with a 32-channel phased-arrayed head coil (parameters: TR/TE/TI = 2000/2.98/900 ms; flip angle = 9°; FOV = 256 × 256 mm^3^; isotropic voxel size = 1 mm^3^). Individual T1-weighted image was preprocessed using Statistical Parametric Mapping 12 (Wellcome Trust Centre for Neuroimaging, London, UK). Images were reoriented to the anterior and posterior axis and segmented to produce native-space GM, WM, and cerebrospinal fluid (CSF) images [58]. Native-space GM images of all participants were then warped and modulated to a study-specific template using a high-dimensional nonlinear diffeomorphic registration algorithm (DARTEL) with the flow field, which contains the information of spatial deformations for normalizing individual images to the DARTEL template [59]. The normalized (to an isotropic 1.5-mm voxel size) and modulated GM images were smoothed with a 4-mm full-width at the half-maximum Gaussian kernel.

### Statistical analysis

#### Mass-univariate approach

Given the imbalanced sample size of the good responder and the poor responder group, nonparametric statistics were employed using the Statistical NonParametric Mapping-13 (SnPM13) toolbox (http://www.fil.ion.ucl.ac.uk/spm/snpm/) [60]. The two groups were compared through a two-sample *t*-test using an approximate test of 20,000 permutations. Results were deemed significant with a cluster-forming voxel-level height threshold of *p* < 0.01 (z > 2.33), and a cluster-level correction for multiple comparisons *p* < 0.05 (familywise error rate). The categorical group variable (good or poor responder to methylphenidate) was used as an independent variable, and sex, total GM volume, age linear and square terms were assigned as nuisance covariates. For the whole-brain hypothesis-free analysis, a mean GM mask was generated using a threshold of > 0.2 in the GM part of the DARTEL template to minimize the contribution of voxels from WM and CSF. According to our hypothesis that the methylphenidate response would be related to GM morphometry of the striatal and DMN regions, we also implemented this nonparametric model restricted within the striatal and DMN masks, respectively (i.e., a small-volume correction). The striatal mask comprising putamen, caudate nucleus, and nucleus accumbens was generated using the Oxford-GSK-Imanova Structural–anatomical Striatal Atlas [61]. The DMN mask was defined using the Yeo-7-network parcellation [62] (Supplementary Fig. S1).

#### Multivariate pattern recognition approach: support vector machine

To complement the preceding mass-univariate analysis, we applied machine learning to test the multivariate pattern differences of GM volumetric images between these two groups using Pattern Recognition for Neuroimaging Toolbox (PRoNTo) version 2.1 [63]. Specifically, Support Vector Machines (SVM) binary classification was implemented by calling the LIBSVM library [35] (v.3.20) to classify good responder group (class 1) and poor responder group (class 2). We employed a whole-brain approach involving the same mean mask to exclude voxels outside the brain GM. Such an approach resulted in feature vectors of 328,062 features (each feature corresponds to a brain voxel). Considering a large number of features, we used a linear kernel, since mapping them to an even higher dimensional feature space with a nonlinear kernel is super expensive and hard to converge. The VBM data were corrected for the effects of sex, age linear and square terms, and total GM volume through a linear regression (all covariates were mean-centered before putting in the model). During the training phase, the two-class SVM algorithm found a hyperplane that separated the examples in the input space, thus maximizing the margin of separation between the class label. Support vectors were data points that juxtaposed closest to the separating hyperplane. When the training data determined the decision function, it could be applied to predict the class label, to which a new test example belonged.

The generalization ability of the model was evaluated using 5-fold cross-validation (CV) in combination with the leave-one-out cross-validation (LOOCV) strategy to ensure that every individual from the dataset had the chance of appearing in the training and test set. Correctly, the dataset was partitioned into five parts at each cross-validation iteration, with 80% of the data to train the model, alongside 20% to test it. LOOCV involved the exclusion of a single instance (i.e., one participant from either good or poor responder) and training the classifier using the remaining subjects in each iteration [64]. LOOCV and k-folds CV have been widely used for classification generalization to avoid overfitting of the model, especially when the sample size is small [64]. The performance of the binary classifiers was evaluated using receiver operating characteristic (ROC) analysis and the area under the ROC curve (AUC). AUC summarized the classification power of a classifier, whereby a classifier with larger AUC indicates its better performance [65]. A 10,000-times nonparametric permutation test by randomly shuffling the labeled class among participants was used to obtain a corrected *p*-value to determine the statistical significance of the accuracy, sensitivity, and specificity. Herein, we adopted the balanced accuracy, as it takes into account the different examples in each class, and gives equal weight to the accuracies obtained on test samples of each class [66].

To increase the interpretability of the multivariate pattern recognition results, we calculated the images summarizing the weights per region of interest (ROI) as defined by the AAL atlas (comprising 116 cortical and subcortical anatomical structures) [67]. The region contributions can be ranked in descending order, yielding a sorted list of regions according to their contribution to the classification model. To investigate the classification power of specific brain ROIs, we computed vector weights. We shortlisted 17 brain regions (top 15%) that show the highest contribution to the classification model of the average of all folds. To further reflect the “reproducibility” of the regions’ ranking across folds, we also computed the Expected Ranking, a measure investigating whether the selected regions are stable across the folds of the cross-validation (i.e., variability in the training data) [68]. The current dataset suffered from a substantial imbalance in sample sizes between the good and poor responder group, which is a potential concern for the sensitivity of the trained classifier. There are a few strategies in the machine learning fields for the imbalanced classification problem. Here we adopted the over-sampling approach [69], i.e., we randomly replicated the samples of the poor responder group during the training and testing processes. However, we guarantee that the same individual does not appear in the training set and testing set at the same time.

## Results

### Sample characteristics

Among 79 participants with ADHD, 63 individuals were allocated in the good responder group, and 16 were in poor responder group based on the CGI-I. There were no significant differences among patients with ADHD with good methylphenidate response and those of poor responders in terms of age, sex, handedness, and IQ profiles, core ADHD symptoms, and ratios of ADHD subtypes (Table 1).

**Table 1:** Demographic and clinical features, alongside global brain volumes between ADHD with good methylphenidate response, ADHD with poor methylphenidate response

Mean (SD)	ADHD with good response (*n* = 63)	ADHD with poor response (*n* = 16)	Statistics *p* value^a^
Age (in years)	17.6 (10.1)	17.1 (8.4)	0.779
Age distribution			0.840 (Fisher’s exact test)
6–10 years old (n, %)	21 (33.3)	4 (25.0)	
11–20 years old (n, %)	22 (34.9)	7 (43.8)	
21–30 years old (n, %)	8 (12.7)	3 (18.8)	
31–40 years old (n, %)	11 (17.5)	2 (12.5)	
42 years old (n, %)	1 (1.6)	0 (0.0)	
Gender, male (n, %)	54 (85.7)	11 (68.8)	0.113
Handedness, right (n, %)	62 (98.4)	15 (93.8)	0.289
Intelligence quotient (IQ)			
Full-scale IQ	107.3 (12.4)	107.8 (10.6)	0.779
Verbal IQ	107.9 (10.0)	106.9 (8.6)	0.779
Performance IQ	105.6 (14.0)	108.4 (13.2)	0.438
Subtype (n, %)			0.666 (Fisher’s exact test)
Inattention type	33 (52.4)	10 (62.5)	
Hyperactivity/impulsivity type	1 (1.6)	0 (0.0)	
Combined type	29 (46.0)	6 (37.5)	
SNAP-IV			
Inattention	17.2 (5.5)	17.7 (5.7)	0.753
Hyperactivity/impulsivity	11.4 (6.6)	10.3 (4.7)	0.610
Opposition-defiance	10.3 (6.2)	7.9 (4.3)	0.166
Inattention and hyperactivity	28.6 (10.7)	27.9 (7.0)	0.985
Total volumes of gray matter (mm^3^)	791.4 (67.1)	798.5 (75.5)	0.626
Total volumes of white matter (mm^3^)	444.5 (57.4)	445.1 (46.6)	0.903
Total volumes of CSF (mm^3^)	276.5 (70.4)	246.9 (53.4)	0.157
Total brain volumes (mm^3^)	1236.0 (82.2)	1243.6 (89.8)	0.600
Total intra-cranial volumes (mm^3^)	1512.4 (122.8)	1490.5 (118.8)	0.634

^a^Mann-Whitney U test, Pearson chi-square test

Abbreviations: *ADHD* Attention Deficit Hyperactivity Disorder, *SNAP-IV* Chinese version of the Swanson, Nolan, and Pelham, Version IV, *CSF* cerebral spinal fluid

### Mass-univariate analysis: whole brain

No significant differences were found in the global brain volume measures, including total GM, WM, and intracranial volume between these two ADHD subgroups (Table 1). The unbiased whole-brain mass-univariate VBM approach yielded no significant difference in regional GM volume between good and poor responders at the aforementioned preset threshold.

### Mass-univariate analysis: small-volume correction

**Fig. 2:**
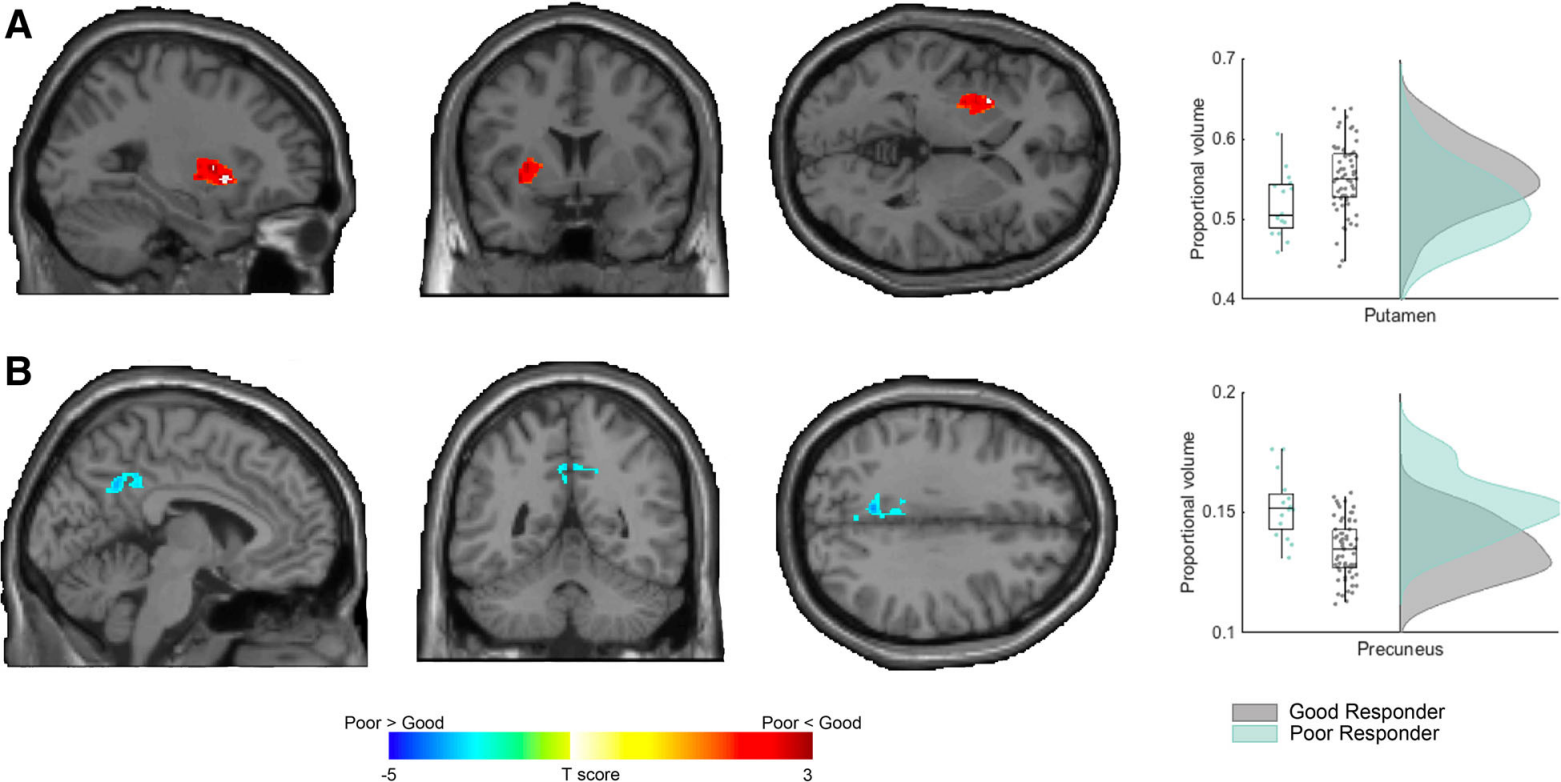
Mass-univariate analysis of relative regional gray matter volumes between participants with ADHD with good and poor methylphenidate response. **a** Using a small-volume correction within the striatum, the good responders had significantly larger GM volumes in the left putamen cluster (1738 mm3, FWE-*p* = 0.032) than the poor responders. **b** Within the DMN mask, the good responders had a significantly smaller GM volume in the bilateral precuneus than the poor responders (3642 mm3, FWE-*p* = 0.012)

Implementing a small-volume correction within the striatum, we identified that the good responders had significantly larger GM volumes in the left putamen cluster (1738 mm^3^, FWE-*p* = 0.032) than the poor responders. Within the DMN mask, significantly smaller GM in the bilateral precuneus was observed in the good responders than the poor responders (3642 mm^3^, FWE-*p* = 0.012) (Fig. 2 & Table 2). In the present study, there was a significant negative correlation (*p* = 0.002, *r =* − 0.349; partial correlation controlling for age, age square, and FIQ) between the volumes of the left putamen and precuneus.

**Table 2:** Significant differences in relative regional gray matter volumes between ADHD participants with good and poor methylphenidate response

Cluster	Region	BA	Hemisphere	MNI coordinates			T value	Cluster-level *p* value^a^	Cluster size (voxels)^b,e^
				x	y	z			
**A**									
**Good responders > Poor responders**									
Left Putamen cluster ^c^									
Putamen_L		–	L	−30	−10	−3	3.84	0.0319	515
**B**									
**Good responders < Poor responders**									
Bilateral Precuneus cluster ^d^									
Precuneus		31	L	−6	−54	36	4.10	0.0115	1079

Abbreviations: *ADHD* Attention Deficit Hyperactivity Disorder, *BA* Brodmann area, *L* Left, *R* Right, *MNI* Montreal Neurological Institute

^a^Regions were identified based on the Harvard-Oxford Atlas

^b^Statistical threshold was all set at FWE-corrected cluster-level *p <* 0.05 (controlled for non-stationarity), with cluster-forming voxel-level *p <* 0.01

^c^A small volume correction within the striatal mask

^d^A small volume correction within the DMN mask

^e^Isotropic voxel size = 1.5 × 1.5 × 1.5 mm^3^

### Post-hoc ROI analysis

To endorse the robustness of the findings, we implemented a post-hoc analysis by using the ROIs of the AAL atlas to extract the regional GM volumes of the left putamen and bilateral precuneus. These ROI GM volumes were divided by the total GM volume to generate proportional volumetric measures. The covariates were then regressed out to eventually yield the GM residual of these ROIs. The nonparametric Mann–Whitney U test revealed the consistent results that individuals in the good responder group, relative to those with poor responses to methylphenidate, had higher regional GM volumes in the left putamen (*p* = 0.010), and smaller volumes in the right (*p* = 0.025) and the left (*p* = 0.031) precuneus. (Table 3).

**Table 3:** Group comparison of different anatomy part of striatal volumes by the approach of voxel-based morphometry

Residual^a^	Demographic and clinical features, alongside global brain volumes between ADHD subgroups	ADHD with poor methylphenidate response (*n* = 16), Mean (SD)	Statistics *p* value
Left Caudate	0.070 (0.994)	−0.290 (0.862)	0.192
Right Caudate	0.103 (0.962)	−0.297 (0.868)	0.118
Left nucleus accumbens	0.114 (1.026)	0.370 (0.873)	0.311
Right nucleus accumbens	0.112 (1.026)	0.374 (0.872)	0.306
Left Putamen	0.117 (0.951)	−0.476 (0.868)	0.010^b^
Right Putamen	0.038 (1.001)	−0.265 (0.985)	0.102
Left Precuneus	−0.138 (0.892)	0.545 (1.174)	0.031 ^b^
Right Precuneus	−0.106 (0.983)	0.416 (0.918)	0.025 ^b^

Abbreviation: *ADHD* Attention Deficit Hyperactivity Disorder

^a^Residual value was done with the independent variables of age and age square

^b^Not surviving Bonferroni correction

### Differentiating two groups by using multivariate pattern recognition approach

**Fig. 3:**
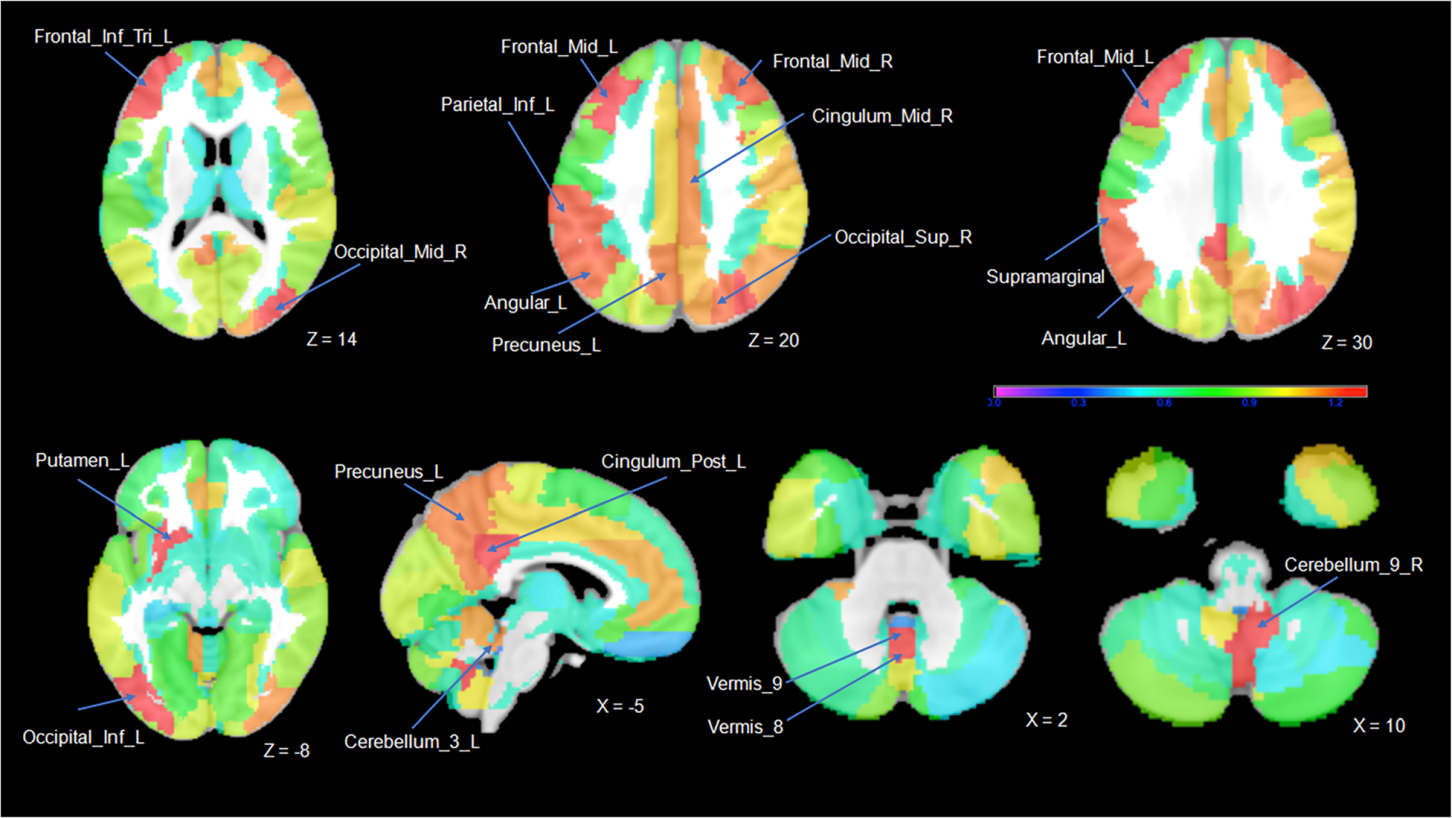
The top 17 areas recognized by machine learning with leave-one-out and 5-folds cross-validation. The bilateral occipital lobes, cerebellar vermis and posterior/inferior cerebellum, posterior cingulate/precuneus, left putamen, and left parietal lobe, and bilateral lateral prefrontal cortex were recognized as the most informative regions for classification between good and poor responders. The color range displayed represents the weight of each ROI, contributing to pattern classification

From the results of the average fold, SVM based on whole-brain analysis differentiated the ADHD-good responder group from the ADHD-poor responder group with 87.4% balanced accuracy (*p* < 0.001). The sensitivity of classification for the good responder group was 93.7%, while the specificity of classification for controls was 81.3%. The positive and negative predictive values for the classifier were 90.8 and 86.7%, respectively. The area under the ROC curve (i.e., AUC) was 0.88 (Supplementary Fig. S2). As shown in Fig. 3, the discrimination weighted ROI map that showed the global patterns that best discriminate good and poor responder groups. For GM VBM features, the most informative regions for classification between good and poor responder groups predominately included the bilateral occipital lobes, cerebellar vermis, and posterior/inferior cerebellum, posterior cingulate/precuneus, left putamen, and left parietal lobe, and bilateral lateral prefrontal cortex. The region’s expected ranking generally corresponds to the ranking in the average fold (Table 4).

**Table 4:** The top 17 areas recognized by machine learning with leave-one-out and 5-folds CV

Index of ROI	ROI Label	ROI weight	ROI Size	Expected Ranking
1	Vermis 9	1.641%	380	2.8
2	Left inferior occipital gyrus	1.513%	1963	1
3	Left posterior cingulate gyrus	1.436%	791	4.4
4	Left middle frontal gyrus	1.374%	4477	4.6
5	Right middle occipital gyrus	1.355%	3283	2
6	Left putamen	1.332%	1637	16.8
7	Right cerebellum 9	1.292%	1162	18.4
8	Left inferior frontal gyrus, triangular part	1.291%	3395	10.2
9	Vermis 8	1.276%	471	20.6
10	Left supramarginal gyrus	1.239%	2505	13.2
11	Left inferior parietal gyrus	1.238%	4444	11.4
12	Right middle frontal gyrus	1.233%	4751	9.4
13	Right superior occipital gyrus	1.219%	1995	8.2
14	Left angular gyrus	1.217%	2321	15.2
15	Left Precuneus	1.188%	5647	14.8
16	Right median cingulate gyrus	1.172%	4432	19.8
17	Left cerebellum 3	1.169%	196	29.4

## Discussion

Using a distinct design in a sample free from confounds of psychiatric comorbidities and medication exposure, we found that participants with ADHD with poor methylphenidate responses clinically had smaller regional volumes in the left putamen and larger precuneus volumes at baseline, compared with the good responders. Multivariate pattern recognition also identified that volumetric information among these two regions alongside the left frontoparietal regions occipital lobes and posterior/inferior cerebellum could differentiate between good and poor responders. This regional structural information, especially striatal volume, if replicated, might serve as a potential biomarker for methylphenidate responses in ADHD.

The finding of greater left putamen volumes in good responders was largely consistent with the prior study showing smaller striatal volumes concentration in individuals with ADHD-poor responders [20]. Several PET studies have demonstrated that methylphenidate could increase striatal dopamine availability [70, 71], and this mechanism is related to the binding to the dopamine transporter [72]. Patients with ADHD with higher striatal dopamine transporter availability show a better response to methylphenidate treatment [15]. Further, striatal dopamine receptor (D2) availability is positively associated with methylphenidate response [73]. In parallel, larger GM volumes are believed to be related to an increased density of neurons and more synapses in the local brain region [74]. The local neural density and the synapses also represent the reciprocal connections within the local brain region and clustering [75]. Furthermore, earlier studies suggest a direct positive correlation between striatal GM volume and D2 receptors [76, 77]. Taken together, the relationship between larger putamen volumes and a good methylphenidate response may be explained by that higher striatal volumes could indicate higher D2 receptor availability locally, which leads to an increase in the methylphenidate efficiency.

To the best of our knowledge, this study is the first work demonstrating the poor responder group, relative to the good responder group, had higher GM volumes of the precuneus, a hub of the DMN [78]. The result echoes earlier evidence that methylphenidate influences DMN activities [30–33] in patients with ADHD. This finding of larger precuneus echoes a similar finding from an earlier PET work. Namely, Tomasi et al. [79] reported that dopamine modulates attention in part by regulating neuronal activity in the posterior parietal cortex, including the precuneus. Higher striatal dopamine transporter levels, which result in enhanced clearance of dopamine and weaker dopamine signals, are associated with lower deactivation in the DMN during an attention-requiring task [79]. This suggests that the DMN deactivation, which should be normally remarkable to facilitate optimal performances during external cognition [80], and could be enhanced by methylphenidate in individuals with ADHD, is mediated by striatal dopamine levels [79]. Taken together, we speculate that lower putamen volumes, as shown in the poor responders, may have lower D2 and dopamine transporter availability, which results in less dopamine increased by methylphenidate. Given negative functional connectivity between precuneus and putamen [81], as well as corticostriatal projections between the precuneus and putamen [82], this lower level of striatal dopamine increase may lead to less dopamine available being used in the precuneus, contributing to less deactivation of the DMN as subserved by methylphenidate. The concomitant larger precuneus volumes in the poor responders might represent a compensatory process for the preceding mechanism. Combining the above mechanisms and our finding of a negative correlation between the volumes of precuneus and putamen, the pattern might be alternatively explained by the notion that structural covariance reflects brain functional connectivity and is resulted from direct structural connections through trophic effects [37]. Specifically, the putamen-associated network [83] has an anti-correlated functional relationship with the DMN/precuneus [27, 81]. Future studies need to replicate the current findings and investigate such speculations.

Although the striatum and DMN were specifically targeted revealed in the univariate VBM analysis in this study, we note that methylphenidate-associated brain structural and functional changes also involve other brain systems and areas, which might be associated with treatment responses as well. For example, methylphenidate has effects on modulating the attention network [84, 85] and normalizes activation of the dorsolateral inferior prefrontal cortex to improve attention [86]. In addition, methylphenidate also could regulate brain activity in premotor cortices [87], which may be associated with its beneficial effects on hyperactivity symptoms. Whether these other putative brain systems/regions are associated with responses to methylphenidate treatment warrants further investigation.

Our finding of the regional brain volume difference between different drug responders by using voxel-based morphometry was also supported by using a machine learning approach, which selected the precuneus and left putamen, endorsing the aforementioned mass-univariate findings. In addition to these two regions, the SVM classification identified that regions of the discriminative pattern most predictive of treatment responses were in the left frontoparietal regions, mid and posterior cingulum gyrus, occipital lobes, as well as posterior cerebellum. There is functional connectivity between the striatum and the posterior cingulate, middle/inferior frontal gyrus [83], and structural connections between the striatum and cerebellum as well as the frontal gyrus, respectively [88]. A human PET study demonstrated that the frontal area and cingulate gyrus are the regions of dopaminergic projection [89]. In macaque monkeys, axons with dopamine transporter are presented in cerebellum lobules III and IX [90]. Norepinephrine transporters distribute not only in high-density regions such as the thalamus and locus coeruleus but also in the low-density regions in the frontal, parietal, and occipital cortex [91]. In these cerebral cortices, norepinephrine transporters are the major transporters for dopamine and norepinephrine reuptake [92]. Altogether, these brain regions selected by multivariate pattern classification contained transporters involving in dopamine and norepinephrine reuptake, which is implicated in methylphenidate mechanisms. Moreover, striatum has direct structural and functional connections with most of these regions [83, 88], which might synergistically contribute to mechanisms underlying responses to methylphenidate with striatum. The finding of the classification accuracy of over 85% based on brain structure measures are promising and, if replicated, suggest that it may be possible in the future to use machine learning-based pattern recognition analyses to aid in the classification of medical response before the application of methylphenidate for patients with ADHD.

Several limitations must be considered while interpreting the results. First, the drug response was evaluated through a retrospective chart review, which consisted of patients’ current progress and detailed medication profile that were sufficient for rating the CGI-I. But there were no details about further cognitive function or the life quality profile. Of note, this study also was limited by a lack of placebo-controlled design. However, this study is a follow-up analysis based on the cohort originally for the cross-sectional study purpose, and the two child psychiatrists who rated the CGI-I were blinded to the participants and their attending psychiatrists. This approach may account for some extents of this caveat. We acknowledge that a prospective longitudinal design may be a more robust approach, e.g., the MTA study [93], to answer such research questions. Second, despite the present “pure” phenotype without confounding effects from psychotropic agents and co-occurring major psychiatric problems, readers need to notice the caveat of generalizability of our results based on such a sample recruited from one medical center in Taiwan. Third, we excluded participants who were lost to follow-up within 1 month of starting using methylphenidate. These patients with ADHD may be more likely to have poor clinical outcomes. However, the percentage of good responders herein was 80%, approximately the ratio of responses to methylphenidate reported before [5], indicating that the current sample was representative of the general ADHD population. Fourth, despite the fact that cerebral morphometric alterations may be different between ADHD subtypes [94], we did not undertake the subgroup analysis based on the subtype, given the limited sample size of the poor responder group. But there was no difference in ratios of subtypes between the good and poor responder groups (Table 1). Future relevant larger studies could benefit from ADHD subtyping analyses. Lastly, the study participants had a relatively wide age range. The mega-analysis using the cross-sectional ENIGMA dataset showed that ADHD had smaller putamen volume in participants with broader age ranges [95]. The altered putamen volume in ADHD was unaltered with age development based on another large NeuroIMAGE sample [96]. To balance the statistical power and difficulty recruiting such a medication-naïve and comorbidity-free sample, we still employed the current sampling approach. The linear and square terms of age was controlled in every model to minimize the confounding effect. However, we acknowledge that some age-related effects may not be excluded statistically. Future studies of a similar kind will need to take developmental issues into account.

## Conclusions

Our findings of the conventional mass-univariate VBM analysis provide evidence that individuals with ADHD having larger precuneus and smaller putamen volumes were more likely to have a poor response to methylphenidate treatment. Such evidence was further confirmed and extended by findings yielded from a multivariate machine learning approach. Our results corroborate the essential role of the striatum in mediating responses to methylphenidate in ADHD [16, 23]. The present study also highlights newly-reported, but not surprising, evidence, indicating the involvement of the DMN in methylphenidate mechanisms [30, 31, 34]. Most of the regions, which were identified to be able to help differentiate clinically good and poor responders, are functionally or structurally linked with striatum. Future studies with larger sample sizes, prospective design, and multimodal MRI measures, should target the striatum and its associated networks to obtain a more comprehensive picture of imaging biomarkers for the prediction of treatment effects of methylphenidate in the ADHD populations.

## Supplementary Information

**Additional file 1: Figure S1.** The masks used to small-volume corrections. Image A shows the masks used to analyze the bilateral striatum in coronal and axial planes. Image B shows the masks used to analyze the default-mode-network in coronal, axial, and sagittal planes.

**Additional file 2: Figure S2.** The area under the receiver operating characteristic curve by machine learning with leave-one-out and 5-folds cross-validation.

**Additional file 3.**

## Data Availability

The datasets generated and/or analyzed during the current study are not publicly available due to confidentiality agreements but are available from the corresponding author on reasonable request.

## Abbreviations

ADHD: Attention-deficit hyperactivity disorder
GM: Gray matter
WM: White matter
CSF: Cerebrospinal fluid
DMN: Default-mode network
VBM: Voxel-based morphometry
NTUH: National Taiwan University Hospital
K-SADS-E: Kiddie Schedule for Affective Disorders and Schizophrenia-Epidemiological version
SNAP-IV: Swanson, Nolan, and Pelham, Version IV
PRoNTo: Pattern Recognition for Neuroimaging Toolbox
SVM: Support Vector Machines
CV: Cross-validation
LOOCV: Leave-one-out cross-validation
ROC: Receiver operating characteristic
AUC: Area under the ROC curve
ROI: Region of interest
CGI-I: Clinical Global Impressions–Improvement Scale
