# Correction to: Regional brain volume predicts response to methylphenidate treatment in individuals with ADHD

**DOI:** 10.1186/s12888-021-03096-3

**Published:** 2021-02-16

**Authors:** Jung-Chi Chang, Hsiang-Yuan Lin, Jinglei Lv, Wen-Yih Issac Tseng, Susan Shur-Fen Gau

**Affiliations:** 1 Department of Psychiatry, National Taiwan University Hospital, Taipei, Taiwan; 2 Department of Psychiatry, National Taiwan University Hospital, Hsin-Chu Branch, Hsin-Chu, Taiwan; 3 Graduate Institute of Clinical Medicine, College of Medicine, National Taiwan University, Taipei, Taiwan; 4 Azrieli Adult Neurodevelopmental Centre and Adult Neurodevelopment and Geriatric Psychiatry Division, Centre for Addiction and Mental Health, Toronto, Ontario Canada; 5 Department of Psychiatry, University of Toronto, Toronto, Ontario Canada; 6 Sydney Imaging and School of Biomedical Engineering, University of Sydney, Camperdown, NSW Australia; 7 Graduate Institute of Brain and Mind Sciences, National Taiwan University College of Medicine, Taipei, Taiwan; 8 Institute of Medical Device and Imaging, National Taiwan University College of Medicine, Taipei, Taiwan; 9 Department of Psychiatry, College of Medicine, National Taiwan University, Taipei, Taiwan

**Correction to: BMC Psychiatry 21, 26 (2021)**

**https://doi.org/10.1186/s12888-021-03040-5**

Following publication of the original article [1], the authors identified an error in the author name of Jinglei Lv.

The incorrect author name is: Junglei Lv

The correct author name is: Jinglei Lv

The author group has been updated above and the original article [1] has been corrected.

## References

[1] Chang (2021). Regional brain volume predicts response to methylphenidate treatment in individuals with ADHD. BMC Psychiatry.

