# Supplementary figures and images for: Regional brain volume predicts response to methylphenidate treatment in individuals with ADHD
Source: BMC Psychiatry. 2021 Jan 11;21:26. doi: 10.1186/s12888-021-03040-5 (PMC7798216; doi:10.1186/s12888-021-03040-5)

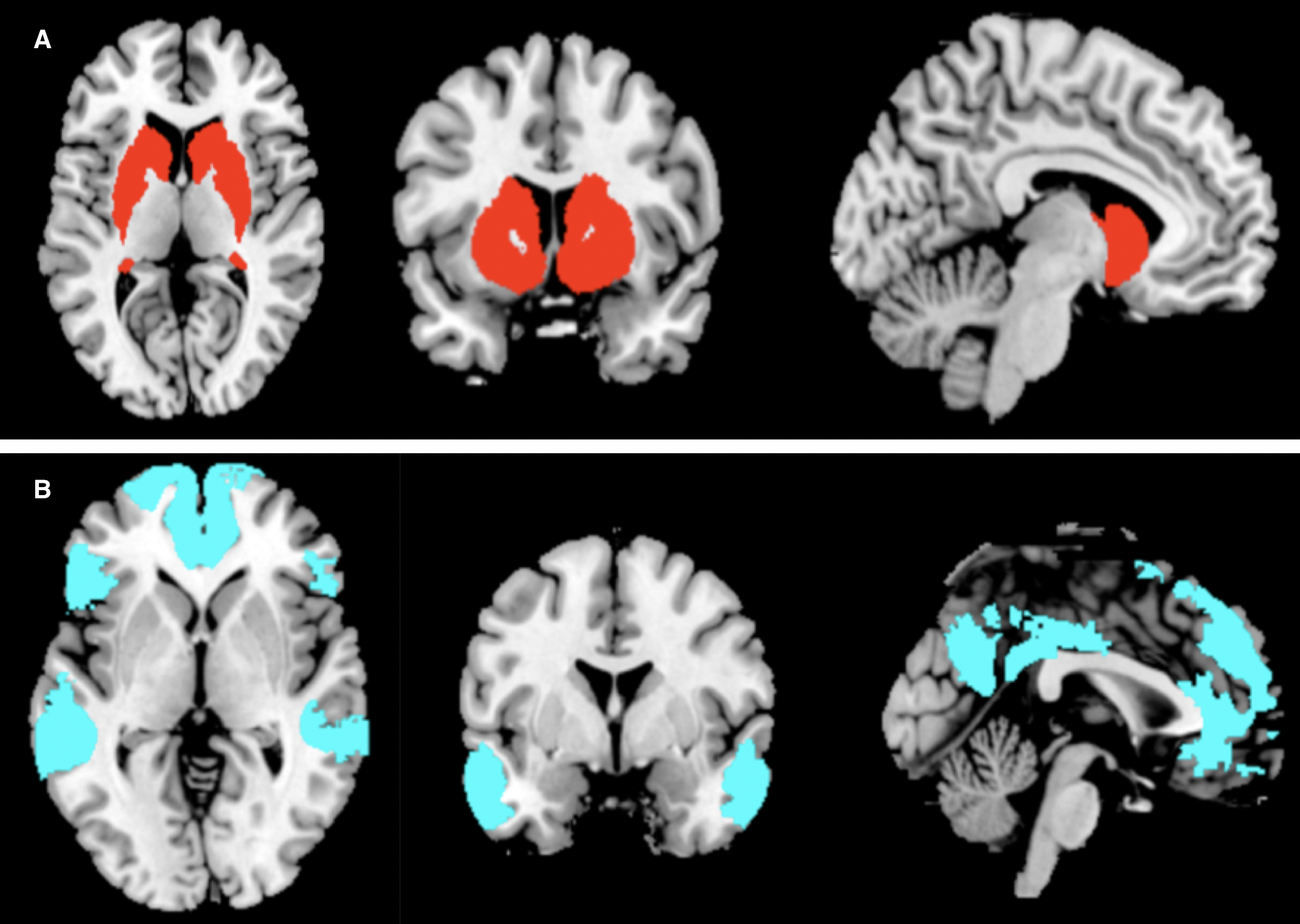

Supplement: Supplementary file 1 — Additional file 1: Figure S1. The masks used to small-volume corrections. Image A shows the masks used to analyze the bilateral striatum in coronal and axial planes. Image B shows the masks used to analyze the default-mode-network in coronal, axial, and sagittal planes. [file 12888_2021_3040_MOESM1_ESM.tiff]

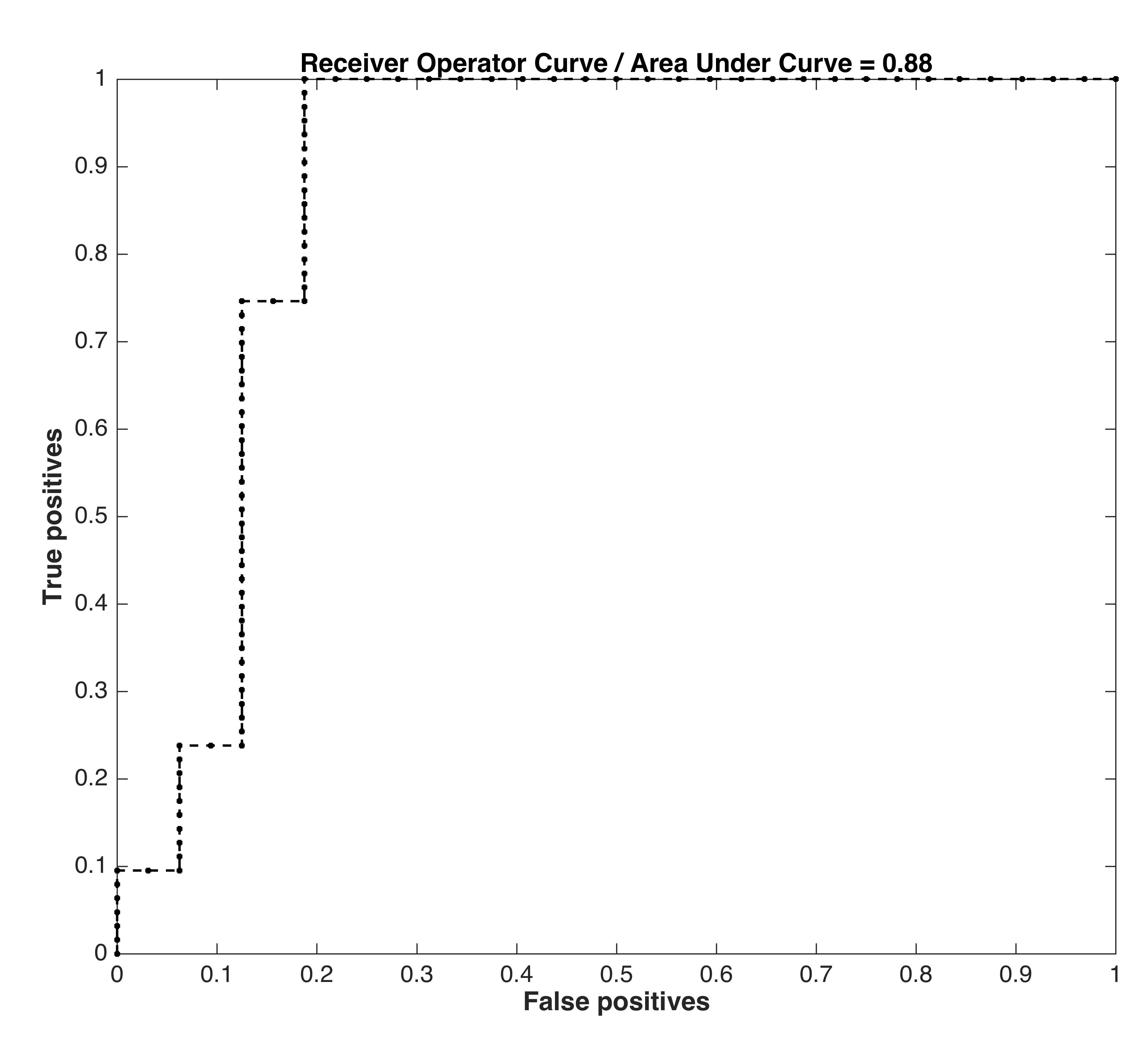

Supplement: Supplementary file 2 — Additional file 2: Figure S2. The area under the receiver operating characteristic curve by machine learning with leave-one-out and 5-folds cross-validation. [file 12888_2021_3040_MOESM2_ESM.tiff]
