# Supplementary material for: Regional brain volume predicts response to methylphenidate treatment in individuals with ADHD
Source: BMC Psychiatry. 2021 Jan 11;21:26. doi: 10.1186/s12888-021-03040-5 (PMC7798216; doi:10.1186/s12888-021-03040-5)
Supplement: Supplementary file 3 — Additional file 3. [file 12888_2021_3040_MOESM3_ESM.pdf]

The SNAP-IV Teacher and Parent Rating Scale

Date Completed:_____/_____/_____ Child ID:__________
Completed by:__________________ □ Parent/Caregiver □ Teacher □ Other, specify__________

Read each item below carefully and check the column that represents your choice; do not mark between two choices. Please be sure to answer every item. In the past week have you noticed that the child….

		Not At All	Just A Little	Quite A Bit	Very Much
1.	Fails to give close attention to details or makes careless mistakes in schoolwork or other activities	_____	_____	_____	_____
2.	Has difficulty sustaining attention in tasks or play activities	_____	_____	_____	_____
3.	Does not seem to listen to what is being said to him or her	_____	_____	_____	_____
4.	Does not follow through on instructions and fails to finish schoolwork or chores (not due to oppositional behavior or failure to understand instructions)	_____	_____	_____	_____
5.	Has difficulty organizing tasks and activities	_____	_____	_____	_____
6.	Avoids, expresses reluctance about, or has difficulties engaging in tasks that require sustained mental effort (such as schoolwork or homework)	_____	_____	_____	_____
7.	Loses things necessary for tasks or activities (eg, school assignments, pencils, books, tools, or toys)	_____	_____	_____	_____
8.	Is easily distracted by extraneous stimuli	_____	_____	_____	_____
9.	Is forgetful in daily activities	_____	_____	_____	_____
10.	Fidgets with hands or feet or squirms in seat	_____	_____	_____	_____
11.	Leaves seat in classroom or in other situations in which remaining seated is expected	_____	_____	_____	_____
12.	Runs about or climbs excessively in situations where it is inappropriate	_____	_____	_____	_____
13.	Has difficulty playing or engaging in leisure activities quietly	_____	_____	_____	_____
14.	Is always "on the go" or acts as if "driven by a motor"	_____	_____	_____	_____
15.	Talks excessively	_____	_____	_____	_____
16.	Blurts out answers to questions before the questions have been completed	_____	_____	_____	_____
17.	Has difficulty waiting in lines or awaiting turn in games or group situations	_____	_____	_____	_____
18.	Interrupts or intrudes on others (eg, butts into other's conversation or games)	_____	_____	_____	_____
19.	Loses temper	_____	_____	_____	_____
20.	Argues with adults	_____	_____	_____	_____
21.	Actively defines or refuses adult requests or rules	_____	_____	_____	_____
22.	Does things deliberately that annoy other people	_____	_____	_____	_____
23.	Blames others for his or her mistakes or misbehavior	_____	_____	_____	_____
24.	Is touchy or easily annoyed by others	_____	_____	_____	_____
25.	Is angry and resentful	_____	_____	_____	_____
26.	Is spiteful or vindictive	_____	_____	_____	_____

The mean of items 1-9 will be summarized for ADHD subset inattention symptoms and the mean of item 10-18 will be summarized for ADHD subset hyperactivity/impulsivity symptoms.
The mean of items 19-26 will be summarized for ODD.
